# Artificial Intelligence for Predicting Postoperative Complications in Orthopedics: A Review of Clinical Applications, Challenges, and Future Directions

**DOI:** 10.7759/cureus.100254

**Published:** 2025-12-28

**Authors:** Aviral C Sharma, Amta Azeem, Ibrahim H Omari, Ajay Premkumar

**Affiliations:** 1 School of Medicine, Ponce Health Sciences University, St. Louis, USA; 2 General Practice, Sheba Medical Center, Ramat Gan, ISR; 3 Department of Orthopaedics, Emory University School of Medicine, Atlanta, USA

**Keywords:** arthroplasty, artificial intelligence, clinical decision support, deep learning, electronic health records, explainable ai, machine learning, orthopedic surgery, postoperative complications, risk prediction

## Abstract

Postoperative complications, including infections, venous thromboembolism (VTE), and prolonged length of stay (LOS), remain major sources of morbidity and healthcare expenditure in orthopedic surgery. While traditional risk stratification tools provide useful benchmarks, they often fall short in delivering precise, individualized predictions. This review extends prior work by providing a direct comparative synthesis of artificial intelligence (AI) and traditional statistical models in orthopedics, while proposing a roadmap of the Validation, Integration, and Regulation (VIR) framework for responsible adoption, emphasizing multicenter validation, workflow-integrated deployment, and appropriate regulatory oversight to support responsible translation. This narrative review synthesizes recent advances in the use of AI and machine learning (ML) models for forecasting postoperative complications in orthopedic surgery. We conducted a structured narrative (non-systematic) review, following SANRA (Scale for the Assessment of Narrative Review Articles) recommendations, of peer-reviewed studies published from January 1, 2017, to July 1, 2025, in PubMed, Scopus, and Google Scholar. Eligible articles involved adult or pediatric orthopedic surgical populations, developed, validated, or applied AI/ML models to predict perioperative or postoperative complications, and reported quantitative performance metrics (e.g., discrimination, calibration, or clinical impact). Imaging-only diagnostic studies, non-orthopedic or non-surgical cohorts, and non-original reports (reviews, editorials, conference abstracts) were excluded. Given heterogeneity in endpoints and validation designs, we performed a structured narrative synthesis without meta-analysis. We also conducted a Prediction model Risk Of Bias ASsessment Tool (PROBAST)-informed, domain-based appraisal for the subset of primary prediction-model studies contributing to the comparative performance synthesis.

AI-driven predictive models often outperform classical statistical methods across outcomes, including prosthetic joint infection, transfusion, implant failure, and nonunion, with reported area under the curve (AUC) values typically in the 0.75-0.90 range for AI/ML models, compared to 0.60-0.75 for traditional regression across the studies reviewed. These comparisons should be interpreted in light of heterogeneity in datasets, endpoints, and validation design, and AUC alone may not capture clinical utility for low-prevalence events without calibration and threshold-based evaluation. Adoption remains constrained by overfitting, limited multicenter validation, inconsistent calibration/utility reporting, explainability, and interoperability challenges. Future work should pursue federated learning, hybrid clinician-AI frameworks, and equity-focused validation to responsibly integrate AI into orthopedic surgical care.

## Introduction and background

Accurate risk prediction is critical in orthopedics to mitigate postoperative complications, including surgical site infections, venous thromboembolism (VTE), hospital readmissions, and prolonged length of stay (LOS). These complications not only increase patient morbidity but also add substantial costs to healthcare systems worldwide [1]. Early identification of high-risk patients allows for tailored perioperative optimization and closer postoperative monitoring, thereby improving outcomes [2]. Since many postoperative events are multifactorial, and some are relatively infrequent, clinically useful prediction tools must balance discrimination (separating high- vs low-risk patients) with actionable insight that can inform perioperative decision-making.

Traditionally, perioperative risk assessment in orthopedics has relied on regression-based tools such as the Charlson Comorbidity Index (CCI) and the American Society of Anesthesiologists (ASA) Physical Status Classification. The CCI incorporates a range of comorbid conditions to estimate 10-year mortality risk, while the ASA score qualitatively stratifies patients based on systemic health [3,4]. However, these models often lack patient-specific granularity, fail to capture complex interactions among multiple risk factors, and generally assume linear relationships that may not exist in real-world clinical scenarios [5,6]. More broadly, many “classical” approaches in this space use statistical models (e.g., logistic regression for binary outcomes) that estimate the independent association between predictors and outcomes under prespecified assumptions (often linearity on the log-odds scale), which supports interpretability but can underfit nonlinear or interactive risk structures unless interactions/nonlinear terms are explicitly engineered.

As a result, there has been a surge in interest in machine learning (ML) and artificial intelligence (AI) approaches that can process high-dimensional data and uncover nonlinear patterns, offering more personalized risk prediction [7-10]. These data-driven models aim to complement clinical judgment and traditional scores by enhancing predictive accuracy and supporting informed decision-making [8,11]. Among commonly used orthopedic ML methods, Random Forest is frequently cited as high-performing; in plain terms, it builds many decision trees on different bootstrap samples of the dataset, restricts candidate predictors considered at each split (random feature selection), and then aggregates predictions across trees (majority vote for classification, averaging for continuous outcomes). This ensemble strategy reduces variance (overfitting to any single tree) while capturing nonlinear relationships and higher-order interactions that standard regression may miss, key reasons performance can differ between Random Forest and classical regression.

In this review, we specifically ask how newer AI-based risk prediction models for postoperative orthopedic complications compare with these traditional regression-based tools in terms of performance and clinical applicability, with the goal of making the discussion accessible to readers who may be less familiar with AI methods. Accordingly, we include a brief, clinician-focused primer on prediction model families so that differences in reported performance can be interpreted in context. For conceptual clarity, we use “AI” as an umbrella term for computational methods that perform tasks typically requiring human intelligence, “ML” as a subset of AI that learns patterns from data rather than relying on hand-coded rules, and “deep learning” as a subset of ML that uses multi-layer neural networks. For clarity, we use the term “high-dimensional data” to describe datasets that contain many variables for each patient (for example, demographics, comorbidities, imaging features, and laboratory values), and “nonlinear interactions” or “nonlinear patterns” to describe situations where the combined effect of multiple risk factors on complications is not simply additive or proportional.

Unlike prior reviews, which primarily catalog emerging AI applications, this article makes three unique contributions. First, it synthesizes comparative evidence from published studies directly comparing AI and classical regression models across multiple orthopedic outcomes, providing quantitative area under the curve (AUC) ranges. Here, the AUC is a standard measure of model discrimination that summarizes how well a prediction tool separates patients who do and do not experience a given postoperative complication, with values closer to 1.0 indicating better performance. Second, it introduces one of the first comparative tables to highlight strengths and limitations side-by-side. Third, it proposes the VIR (Validation, Integration, Regulation) framework as a structured roadmap for responsible adoption. Together, these contributions position this review as a novel, pragmatic, and actionable resource for the orthopedic community. In plain terms, this review (i) summarizes how well AI models perform relative to familiar regression-based scores, (ii) presents a side-by-side comparison to clarify where each approach excels or falls short, and (iii) offers a practical VIR framework that clinicians can use when evaluating or implementing AI tools in everyday orthopedic practice. To support non-technical readers, we emphasize that “better AUC” alone is insufficient for adoption; robust external validation, calibration, and workflow integration are essential to ensure models generalize beyond the development dataset.

Types of prediction models

Classical Statistical Models

Classical statistical methods remain foundational in orthopedic outcome prediction, providing the backbone for much of the historical literature on perioperative risk. Logistic regression is widely employed for modeling binary outcomes, such as the occurrence of surgical site infections (SSI) or the presence of postoperative complications [12,13]. Its output, odds ratios, offers straightforward interpretability, allowing clinicians to quantify how changes in patient characteristics impact event likelihood. In logistic regression, effects are typically modeled as linear contributions to the log-odds of the outcome; as a result, nonlinear relationships and interactions may be missed unless explicitly specified.

For time-to-event analyses, such as evaluating implant survival or time to revision following joint arthroplasty or spinal instrumentation, Cox proportional hazards models are the preferred tool. These models estimate hazard ratios, offering insight into how risk evolves over time [14,15]. Cox models additionally assume proportional hazards over time, which may not hold in all postoperative trajectories (e.g., early vs late failure mechanisms).

The enduring appeal of these classical approaches lies in their transparency and ease of interpretation, making them accessible to both clinicians and researchers. Their mathematical assumptions are explicit, and model coefficients are directly linked to outcome changes, which supports clinical intuition and informed decision-making [16,17]. This interpretability can also facilitate bedside communication and shared decision-making because clinicians can explain how specific covariates influence estimated risk.

However, this interpretability comes with notable limitations. Logistic regression and Cox models generally rely on assumptions of linearity and proportional hazards, respectively [18]. They also struggle to fully capture complex, nonlinear interactions between multiple risk factors, particularly in heterogeneous surgical populations characterized by varied comorbidities and operative nuances [18,19]. As a result, their predictive performance may be suboptimal when faced with intricate real-world datasets, where ML approaches have demonstrated superior ability to model such complexities [19]. Importantly, even when discrimination is acceptable, classical models can still exhibit miscalibration in new settings (systematically over-or under-estimating absolute risk), underscoring the need for external validation and calibration assessment regardless of model class.

Tree-Based Ensemble Models for Tabular Orthopedic Data: Random Forest and Gradient Boosting

In contrast, ML approaches such as Random Forest and gradient boosting machines are nonparametric, allowing them to uncover nonlinear relationships and higher-order interactions that may be missed in classical models [20-22]. Importantly, “ML” is not a single approach, and the most commonly used orthopedic risk-prediction algorithms differ in how they learn from tabular perioperative data. Random forest averages many decorrelated decision trees trained on bootstrapped samples, which reduces variance and can be relatively robust to noisy predictors and mixed variable types commonly seen in electronic health records (EHRs)/registry datasets.

Gradient boosting algorithms (e.g., XGBoost/LightGBM) iteratively add trees to correct prior errors, which often yields strong discrimination on structured perioperative data but increases sensitivity to tuning choices (learning rate, depth, regularization) and therefore increases the risk of overfitting if validation is weak [21].

Neural Networks and Multimodal Deep Learning

Deep learning models can learn hierarchical representations and may integrate structured EHR variables, imaging, and unstructured clinical text. In orthopedics, neural-network approaches are most defensible when the input is genuinely high-dimensional (e.g., imaging or multimodal signals) and when sample size and regularization are sufficient to prevent overfitting. Because deep learning models can be less transparent, studies intended for bedside translation should pair them with interpretability strategies (e.g., feature attribution for tabular data or saliency methods for imaging) and should report calibration to support safe decision-making [7-10]. 

For these reasons, differences in reported performance across studies may reflect not only “AI vs regression,” but also whether the algorithm family matches the underlying data type, sample size, complication prevalence, and validation rigor. Random forest, specifically, is an ensemble of decision trees: each tree is trained on a bootstrapped sample, each split considers only a random subset of predictors, and final predictions are aggregated across trees. Compared with a single decision tree, this aggregation reduces variance and improves generalization; compared with logistic regression, it can learn nonlinearities and interactions without explicitly predefining them.

Numerous studies in orthopedics have demonstrated that these ML methods often achieve superior predictive performance compared to traditional regression, reflected in higher AUC values and, when reported, sometimes improved calibration across diverse patient cohorts [20,23-25]. Their flexibility in modeling complex data structures makes them particularly well-suited for risk stratification in multifaceted clinical scenarios, such as predicting complications after total joint arthroplasty or spinal fusion [26-28]. However, higher AUC in development cohorts may reflect overfitting if models are not rigorously validated; therefore, transparent reporting of internal validation (e.g., cross-validation/bootstrapping), external validation, and calibration metrics is essential when comparing ML to regression.

Building on these advances, deep learning, particularly through neural networks, further extends the capabilities of ML by autonomously learning hierarchical representations of data [29]. These models excel at integrating diverse data types, including structured electronic health record variables, radiographic imaging, and even unstructured clinical narratives such as operative notes or pathology reports [30,31]. As a result, deep learning offers a path toward truly multimodal predictive frameworks that can harness the full spectrum of perioperative information [32-34]. Since deep learning models can be less transparent, studies increasingly pair them with interpretability approaches (e.g., saliency methods for imaging or feature-attribution techniques such as SHAP for tabular data) to support clinical trust and adoption.

By leveraging these sophisticated models, the field is moving closer to achieving personalized risk prediction, enabling tailored surgical planning, more precise intraoperative decision-making, and enhanced patient counseling grounded in comprehensive, data-driven insights [35-37]. In this review, we interpret reported performance differences through this lens: model flexibility (nonlinearity/interactions), validation rigor, calibration, and interpretability/workflow feasibility.

Why Models Perform Differently in Orthopedic Datasets

Differences in reported performance across “Random Forest vs logistic regression vs neural networks” are rarely due to an “AI label” alone. In orthopedics, model performance is strongly shaped by the match between algorithm family and data characteristics, particularly: (i) predominantly tabular EHR/registry features (mixed data types, missingness), (ii) relatively low event rates for many complications (class imbalance), (iii) moderate sample sizes at single centers, (iv) institution-specific workflows that can induce spurious correlations, and (v) the validation design (random split vs temporal split vs external validation).

Logistic regression (and Cox models) typically perform best when relationships are approximately linear (or can be made linear with prespecified transformations) and when interpretability and calibration are prioritized for bedside counseling. They can underperform when complication risk depends on nonlinear interactions (e.g., comorbidity combinations, operative-time thresholds, lab-value interactions) unless these interactions are explicitly engineered.

Random Forest often performs well on tabular perioperative data because they automatically capture nonlinearities and interactions and reduces overfitting by averaging many decorrelated trees. However, Random Forest performance can plateau when optimal decision boundaries require sequential “error-correction,” and it can still overfit when event counts are low or when hyperparameters are not tuned with robust validation.

Gradient boosting (e.g., XGBoost/LightGBM) frequently achieves stronger discrimination than Random Forest on structured EHR/registry datasets because boosting iteratively adds trees to correct prior errors. These gains are most credible when studies report careful tuning, class-imbalance handling, and external or temporal validation; otherwise, boosted models can produce optimistic AUCs that do not generalize.

Neural networks tend to be most advantageous when orthopedic datasets include high-dimensional or multimodal inputs (imaging + structured variables + clinical text). In smaller single-center tabular datasets, neural networks may not outperform boosting/regression due to sample-size demands and overfitting risk; additionally, their “black-box” nature can limit clinical trust unless paired with explainability methods and clear calibration reporting.

Accordingly, throughout this review, we interpret comparative AUCs through an algorithm- and methodology-aware lens: the “best” model family depends on data modality, event prevalence, sample size, missingness, and, most importantly, validation rigor and calibration, rather than on whether a model is labeled “AI.”

Current applications in orthopedics

Total Joint Arthroplasty: Predicting Infection, Transfusion, Length of Stay, and Readmission

One of the most robust and rapidly evolving domains for AI and ML integration is total joint arthroplasty (TJA), encompassing both total hip arthroplasty (THA) and total knee arthroplasty (TKA) [38]. Given the high procedural volume and significant costs associated with these interventions, optimizing perioperative outcomes is a major priority in orthopedic surgery [26,39].

AI-driven models have been developed to predict a wide range of outcomes, including prosthetic joint infection (PJI), perioperative transfusion requirements, LOS, and discharge disposition following hip and knee arthroplasty [40-42]. Importantly, most arthroplasty prediction studies rely on structured EHR/registry variables, making these primarily tabular-data problems; in this context, performance differences often reflect how well each model family captures nonlinear relationships/interactions and how rigorously it is validated, rather than the “AI” label alone. 

For example, ML algorithms such as Random Forests, support vector machines, and neural networks have been applied to identify patients at elevated risk for PJI, one of the most devastating complications after arthroplasty [43-46]. Early identification allows surgeons to implement tailored prophylaxis protocols and intensified postoperative surveillance [46]. Since PJI is relatively uncommon, AUC alone can overstate clinical utility; a model may appear strong on discrimination while still producing modest positive predictive value, making calibration and threshold-based performance (sensitivity/specificity at clinically relevant cutoffs) essential for interpretation. Where available, studies should also report decision-focused measures (e.g., decision-curve net benefit) to clarify whether predicted risk meaningfully changes management.

Similarly, predictive models for perioperative transfusion requirements leverage dozens of preoperative and intraoperative variables to achieve superior accuracy compared to traditional logistic regression, enabling better planning for blood management strategies [47,48]. AI models have also been extensively used to forecast LOS and discharge needs, assisting hospitals in resource allocation and initiating early case management, which may reduce readmission rates [49-52]. Notably, these models frequently outperform traditional regression methods in receiver operating characteristic (ROC) analyses, often achieving AUC values exceeding 0.85, thus offering improved stratification of high-risk patients [40-42]. In many TJA cohorts, tree-based ensembles (e.g., gradient boosting and Random Forest) are particularly well suited to mixed perioperative variables because they can learn nonlinear patterns without extensive manual feature engineering, yet these gains are most credible when studies report robust internal validation (e.g., cross-validation/bootstrapping) and, ideally, external or temporal validation. As a result, AI-enhanced predictive tools are being integrated into perioperative dashboards, supporting clinical decision-making and facilitating individualized care pathways [53,54]. 

Several studies have also used ML to predict 30-day readmission after arthroplasty, reporting performance that exceeds logistic regression and enabling earlier discharge planning and targeted follow-up for high-risk patients [49-52]. Given that readmission risk can be strongly influenced by institutional practice patterns and care pathways, external or temporal validation is particularly important to distinguish generalizable risk signals from site-specific workflows.

In total joint arthroplasty, tree-based ensembles (Random Forest/boosting) frequently performed well on structured perioperative variables (demographics, comorbidities, labs, operative factors), whereas neural-network approaches were most useful when multimodal inputs (e.g., imaging or free text) were included. Reported gains were most credible when paired with temporal or external validation and calibration reporting; otherwise, higher AUC may reflect single-center optimism or class imbalance.

Spine Surgery: Predicting Hardware Failure, Infection, and Reoperation

Similarly, in spine surgery, ML tools have been developed to predict complications such as surgical site infection, hardware failure, pseudoarthrosis, and unplanned revision or reoperation [55-57]. These outcomes carry substantial morbidity and cost, and accurate risk estimation may help guide perioperative optimization, surveillance, and counseling in complex spine populations [56,58]. Compared with arthroplasty, spine cohorts are often more heterogeneous in pathology and procedure type, which can inflate apparent performance in single-center datasets and reduce transportability across institutions.

ML models are being used preoperatively to identify patients at higher risk of instrumentation-related complications such as rod fractures, cage subsidence, or screw loosening [59,60]. This enables surgeons to consider alternative constructs, optimize bone health preoperatively, or implement enhanced follow-up protocols, ultimately aiming to reduce the need for costly and morbid revision surgeries [59]. This is a setting where “model choice” should be explicitly justified: if inputs are primarily tabular, ensemble methods may be the most data-efficient and generalizable; if imaging adds a truly independent predictive signal, multimodal approaches may be appropriate, but only when sample size, preprocessing, and validation rigor support higher model capacity.

Across studies, reported performance improvements should be interpreted in the context of validation design and reporting completeness. Neural-network approaches may appear superior in small internal cohorts yet fail to generalize without strong regularization and external validation; conversely, well-constructed regression or ensemble models can remain highly competitive when predictors are clinically grounded, and calibration is carefully assessed.

ML has also shown promise in predicting surgical site infections (SSI) after spine procedures, incorporating a wide array of demographic, comorbidity, and operative variables that traditional models may not capture as effectively [61-63]. Moreover, predictive models assessing reoperation risks within defined postoperative windows (e.g., 30 days or one year) can support shared decision-making, offering patients individualized risk assessments that guide realistic expectations [64-66]. 

Beyond risk prediction, these models are being integrated into tools that tailor perioperative planning, suggest optimal surgical strategies, and even provide intraoperative decision support by adapting to real-time data streams [67,68]. Importantly, such platforms facilitate more informed patient counseling, leveraging personalized risk data to guide discussions around treatment options and postoperative expectations [69,70].

In spine surgery, performance differences across Random Forest, boosting, and neural networks appeared closely tied to feature type (EHR/registry vs multimodal), low event rates (e.g., SSI/reoperation), and validation rigor. Tree-based ensembles were commonly favored for tabular perioperative data, but their apparent advantage was strongest in studies reporting robust tuning, class-imbalance handling, and temporal or external validation with calibration.

Trauma and Fracture Care: Predicting Mortality, Infection, and Recovery Outcomes

AI has increasingly been utilized in trauma and fracture care to predict postoperative complications, enhancing preoperative risk stratification and personalized management. Accordingly, performance differences across model families often reflect real data constraints, class imbalance for rare complications, missingness in acute settings, and evolving clinical trajectories, rather than inherent superiority of a given algorithm. A retrospective study of 1,720 femoral shaft fracture patients in 2025 demonstrated that an XGBoost model achieved an AUC of 0.83 in predicting 30-day mortality, outperforming traditional logistic regression and identifying key predictors such as age, preoperative white blood cell count, and creatinine levels [71]. The authors emphasized that their study was the first to internally validate an AI-based model capable of predicting 30-day postoperative death rate specifically in patients with isolated femoral shaft fractures. Studies like these highlight how AI is capable of processing multiple clinical variables to predict serious adverse events, allowing physicians to intervene early.

ML approaches can be valuable when they integrate multiple risk domains (injury severity, physiologic derangement, comorbidities, and care-process variables) and account for nonlinear relationships that occur in acute care environments. However, for low-prevalence complications, high AUC can coexist with limited positive predictive value; models should therefore be evaluated using calibration and clinically actionable thresholds, ideally tied to explicit decisions (e.g., ICU triage, enhanced monitoring, infection surveillance intensity).

Beyond discrimination, the most clinically meaningful studies are those that demonstrate transportability across systems and minimize unintended harm. Because trauma populations can differ substantially across regions and institutions, robust evaluation across sites (or temporal validation approximating deployment) is essential to reduce the risk of spurious correlations and to avoid amplifying disparities in access to resources or escalation of care.

In polytrauma patients with concomitant traumatic brain injury (TBI) and fractures, ML techniques have been used to identify optimal surgical timing to minimize postoperative infection risk [72,73]. A 2025 study utilizing Random Forest, XGBoost, and other algorithms achieved an AUC-ROC of 0.84 for predicting infection and an accuracy of 0.81 in validation [72]. The authors concluded that ML models accurately predict optimal surgical timing and conditions, which may reduce postoperative infections and enhance patient outcomes. These models are capable of integrating complex patient data such as Glasgow Coma Scale, hemoglobin, and D-dimer levels, which clinicians can use to guide decision-making, representing a pivotal step towards personalized trauma care. 

Across arthroplasty, spine, and trauma, the most compelling studies are those that pair appropriate model capacity with rigorous temporal/external validation, calibration, and explicit linkage of predictions to actionable decisions, because transportability and clinical utility (not AUC alone) determine whether these tools improve outcomes at the bedside [74,75].

In trauma and fracture care, gradient boosting and other tree-based models often yielded strong discrimination using time-sensitive physiologic and laboratory variables plus injury-severity features, while neural networks may add value when imaging or longitudinal signals are incorporated. Because complication risk is highly time- and site-dependent, temporal and external validation (with calibration and threshold-based clinical utility analyses) is essential before clinical deployment.

## Review

Methods

Search Strategy

We performed a narrative (non-systematic) review of the current literature by searching PubMed, Scopus, and Google Scholar for studies published between January 1, 2017, and July 1, 2025. This narrative review followed the SANRA (Scale for the Assessment of Narrative Review Articles) recommendations to clearly define the aims, transparently report the literature search, and ensure accurate referencing. The search window and date limits were prespecified before screening (January 1, 2017-July 1, 2025). For PubMed, the core Boolean search string was (“artificial intelligence” OR “machine learning”) AND (orthopedic OR orthopaedic) AND (surgery OR surgical) AND (“postoperative complications” OR “risk prediction” OR “prognostic model”); database-specific adaptations were used for Scopus and Google Scholar, and searches were limited to human studies published in English. Search terms included combinations of “artificial intelligence”, “machine learning”, “orthopedic surgery”, “risk prediction”, and “postoperative complications”.

Eligibility Criteria

We included peer-reviewed original research articles that (i) involved adult or pediatric orthopedic surgical populations, (ii) developed, validated, or applied an AI/ML model to predict perioperative or postoperative complications, and (iii) reported at least one quantitative outcome such as discrimination, calibration, or clinical impact. When calibration or clinical impact metrics were not reported, we documented these as reporting gaps. Since discrimination (AUC) was the most consistently reported metric across studies, we used it as the primary summary measure for comparing AI/ML and regression-based models. However, AUC alone does not capture clinically important aspects of performance such as sensitivity, specificity, predictive values, F1 score, or calibration, particularly when predicting rare complications. Studies were excluded if they (i) focused solely on imaging diagnosis without perioperative or postoperative risk prediction, (ii) were case reports, narrative commentaries, conference abstracts, or editorials, (ii) did not report original data (e.g., reviews or opinion pieces), or (iv) were non-human or non-orthopedic. Titles and abstracts identified by the search were screened for eligibility by the first author; potentially relevant full texts were reviewed in detail, and uncertainties regarding inclusion were resolved through discussion with co-authors until consensus was reached. Reference lists of included articles and key reviews were manually searched to identify additional eligible studies.

Screening, Data Extraction, and Analysis

Since the included studies were heterogeneous in outcome definitions (e.g., infection vs readmission vs nonunion), predictors (EHR/registry features vs imaging/multimodal inputs), populations (procedure types and case-mix), and validation designs (random split vs cross-validation vs temporal/external validation), we did not perform a meta-analysis, meta-regression, or pooled effect-size estimation. Instead, we conducted a structured narrative synthesis focused on transparent, within-study reporting of model performance and evaluation design. 

We prespecified discrimination as the primary performance domain (AUC/area under the ROC (AUROC)/C-statistic) because it was the most consistently reported across studies; however, we treated AUC as an incomplete measure of bedside value, particularly for low-prevalence complications. Therefore, when available, we also extracted and summarized: (i) calibration (e.g., calibration plots, calibration intercept/slope, Brier score, observed-to-expected ratios), and (ii) clinical utility/threshold-based performance (e.g., sensitivity, specificity, positive predictive value (PPV)/negative predictive value (NPV), F1 score, and decision-curve analysis/net benefit). When these secondary metrics were not reported, we explicitly noted them as reporting gaps and avoided inferring “clinical usefulness” from AUC alone.

In addition to extracting headline discrimination metrics (primarily AUC), we abstracted study-level details needed to interpret why a given model performed well or poorly, including (when reported): input feature domains (demographics/comorbidities/labs/imaging/text), preprocessing and missing-data handling, management of class imbalance for rare complications (e.g., reweighting or resampling), feature selection/regularization, hyperparameter tuning approach, validation design (train-test split vs k-fold cross-validation vs temporal split), presence and type of external validation, and reporting of calibration or clinical utility (e.g., calibration plots, Brier score, decision-curve analysis). We prioritized interpretation of performance claims using this evaluation context (event rate, validation rigor, and calibration/utility reporting), rather than attributing differences to an algorithm label alone. When comparing AI/ML models to regression, we treated “outperformance” as a within-study finding and reported it only when the comparison was conducted under the same dataset and validation design. Across-study comparisons were described as ranges and trends rather than definitive superiority statements, because AUC values are not directly comparable across different endpoints, prevalence, and validation frameworks.

Eligible studies focused on the development, validation, or clinical application of AI or ML models aimed at predicting perioperative or postoperative outcomes in orthopedic populations. Particular emphasis was placed on models predicting surgical site infections, implant failure, transfusion requirements, nonunion, and hospital readmissions. To reduce optimism bias and provide a balanced overview, we included studies regardless of whether AI/ML models outperformed, matched, or underperformed traditional regression-based methods, and we retained neutral or negative results when identified. Following full-text review, we also re-evaluated all candidate references and removed reports that did not directly examine AI/ML-based prediction of postoperative orthopedic complications, ensuring that each remaining citation is closely linked to the specific claim or outcome it supports. 

Additionally, we performed a Prediction model Risk Of Bias ASsessment Tool (PROBAST)-informed appraisal for the subset of peer-reviewed, primary prediction-model studies that directly contributed to the comparative performance synthesis and the domain-based appraisal summary. Specifically, we extracted and summarized PROBAST-relevant elements across the four domains (Participants, Predictors, Outcome, and Analysis), with emphasis on validation design (random split/cross-validation vs temporal/external validation), handling of missing data (complete-case exclusion vs imputation), management of class imbalance for rare complications (e.g., resampling/weighting where reported), and reporting of calibration and clinical utility (e.g., calibration plots or intercept/slope, Brier score, decision-curve analysis). Given the narrative design and heterogeneity in endpoints and reporting, we used PROBAST as a structured, domain-level qualitative framework rather than generating numeric scores; when key methodological details were not reported, we conservatively recorded them as “not reported/unclear” and interpreted comparative performance findings accordingly.

Results

Comparative Evidence Table

To better illustrate the relative advantages of AI, we synthesized findings across orthopedic outcomes into a comparative framework as summarized in Table 1. Across the primary prediction studies contributing to Table 1, risk of bias was most commonly driven by the analysis domain (single-institution development, limited external validation, and inconsistent calibration reporting). Traditional regression-based models, such as logistic and Cox regression, generally demonstrated modest discriminatory ability, with representative orthopedic prediction studies published between 2017 and 2025 reporting AUC values in the 0.60-0.75 range for outcomes such as surgical site infection, transfusion, LOS, and implant survival [13,40,47,61,62,71]. In contrast, AI and ML methods, including Random Forest, gradient boosting, support vector machines, and neural networks, typically achieved higher AUC values (approximately 0.75-0.90) across arthroplasty, spine, and trauma applications in the same time window [24-27,40,41,47,55-60,61-63,71,72]. This performance gap was most pronounced for implant failure and nonunion prediction, where deep learning-based models reported AUC improvements of more than 0.10 compared to Cox regression in spine fusion cohorts [59,60]. Similarly, perioperative transfusion requirements and postoperative infections showed superior predictive accuracy with ML algorithms in several arthroplasty, spine, and trauma datasets [40,41,47,61-63,71,72]. These comparisons are primarily based on discrimination (AUC), as other performance metrics such as sensitivity, specificity, predictive values, and calibration were reported inconsistently across studies. 

**Table 1:** Comparative performance of traditional regression-based models versus AI/ML models for predicting postoperative orthopedic complications. Values represent AUC ranges reported in primary orthopedic prediction studies published between 2017 and 2025 that directly evaluated postoperative complications after orthopedic surgery (e.g., surgical site infection, transfusion, nonunion, implant failure, readmission, and length of stay). Regression performance ranges were extracted from logistic or Cox models, whereas AI/ML performance ranges were extracted from Random Forest, gradient boosting, support vector machines, neural networks, or ensemble approaches. To minimize citation clustering, we limited the supporting references for this table to clinical studies that explicitly report AUCs for the outcomes listed (e.g., [13,40,41,47,57,59-62,71,72]). The performance ranges therefore incorporate both studies in which AI/ML clearly outperformed regression models and studies in which AI/ML performance was similar or inferior, rather than only reporting best-case AI results. AUC ranges are synthesized from peer-reviewed studies included in this review. AI: artificial intelligence; ML: machine learning; AUC: area under the curve; ANN: artificial neural network; SVM: support vector machine; RF: Random Forest; CCI: Charlson Comorbidity Index; ASA: American Society of Anesthesiologists

Outcome	Traditional Models (e.g., Logistic/Cox Regression)	Typical AUC Range	AI/ML Models (e.g., RF, XGBoost, ANN)	Typical AUC Range	Key Limitations
Surgical Site Infection	Logistic regression, nomograms	0.65-0.75	Random forest, ANN, SVM	0.80-0.88	Traditional: linearity assumptions; AI: overfitting, explainability
Length of Stay (LOS)	Regression-based LOS predictors	0.60-0.72	Gradient boosting, ensemble ML	0.78-0.85	Traditional: weak calibration; AI: limited multicenter validation
Blood Transfusion	Logistic regression	0.68-0.74	ML classifiers (XGBoost, RF)	0.80-0.87	Traditional: fewer variables; AI: requires large, clean datasets
Implant Failure/Nonunion	Cox proportional hazards models	0.62-0.70	Deep learning survival analysis	0.81-0.90	Traditional: poor nonlinear capture; AI: limited interpretability
Readmission	Logistic regression, CCI, ASA-based scores	0.65-0.73	Neural networks, ensemble ML	0.80-0.86	Traditional: coarse stratification; AI: dataset bias concerns

Importantly, we did not interpret AUC as sufficient evidence of bedside utility. When studies reported calibration (e.g., calibration plots, Brier score, observed vs expected ratio (O:E)) or decision/utility metrics (e.g., sensitivity/specificity at clinically relevant thresholds, PPV/NPV, F1 score, or decision-curve net benefit), these were extracted and summarized alongside AUC to contextualize whether discrimination translated into accurate absolute risk estimation and actionable decision support. However, because calibration and decision-curve/utility reporting were inconsistent across the orthopedic AI literature, we did not attempt pooled quantitative comparisons of these secondary metrics; instead, we highlight them as key reporting gaps and interpret “outperformance” claims cautiously, particularly for low-prevalence complications where high AUC can coexist with modest PPV.

To avoid “AI” being treated as a monolith, we also interpreted performance ranges by separating (i) tree-based ensembles (Random Forest/gradient boosting) from (ii) neural-network approaches, because these families have different strengths and failure modes in postoperative complication prediction. In the reviewed orthopedic studies that relied primarily on structured perioperative variables (tabular EHR/registry features), boosted tree models were most frequently associated with higher AUCs, plausibly because they handle mixed data types, nonlinear interactions, and limited feature engineering efficiently; however, these gains were most credible when paired with rigorous tuning and external validation. Conversely, neural-network approaches were most compelling when studies incorporated higher-dimensional inputs (e.g., imaging or multimodal data streams), but their interpretability and calibration reporting were often less consistent, which are limitations that directly affect bedside usability. Accordingly, when two studies reported similar AUCs despite different algorithms, we prioritized methodological explanations (validation design, event rate/class imbalance handling, and calibration/clinical utility reporting) rather than attributing differences to the algorithm label alone. 

During revision, we re-reviewed all references cited in this comparative section and removed methodological, non-orthopedic oncology, and narrative review articles, so that the citations now point only to primary orthopedic prediction studies that report AUC values for the outcomes summarized in Table 1. Nonetheless, the added performance of AI comes with limitations, including risks of overfitting in single-institution datasets, limited generalizability across diverse patient populations, and reduced interpretability compared to regression-based models [9,76]. By presenting summarized performance ranges with key strengths and weaknesses, Table 1 highlights both the promise and current barriers of AI integration into orthopedic postoperative risk prediction.

PROBAST-Informed Study Quality/Applicability Summary

Across the subset of primary prediction-model studies contributing to our comparative performance synthesis (Table 1) and PROBAST-informed appraisal (Table 2), the most recurrent threats to validity were concentrated in the Analysis domain, particularly the predominance of internal validation strategies (random train-test splits and/or k-fold cross-validation) with less frequent temporal or external validation, which can inflate reported discrimination when models are tested on data closely resembling the development cohort. Reporting of missing data handling and class imbalance management also varied substantially across studies, with several using complete case exclusions and others reporting explicit imputation and/or resampling approaches for rare outcomes. In addition, although some studies reported calibration and/or clinical utility metrics (e.g., calibration plots, Brier score, decision-curve analysis), these were not uniformly presented, limiting direct comparability of bedside “usefulness” across algorithms. Accordingly, we interpreted AUC differences cautiously and emphasized validation rigor, calibration, and threshold-based performance when discussing comparative findings rather than attributing differences to an algorithm label alone. This assessment was intentionally PROBAST-informed and domain-based (qualitative), rather than a full PROBAST signaling-question adjudication with overall low/high/unclear risk-of-bias ratings, given heterogeneity in endpoints and reporting across included studies.

**Table 2:** PROBAST-informed appraisal of primary prediction model studies contributing to Table 1. Items are recorded as “Yes/No/NR/Unclear” based on study reporting; when methodological details (e.g., missing-data handling, class imbalance handling, calibration/utility) were not reported, they are conservatively coded as “NR/Unclear.” This appraisal is intended to contextualize AUC-based performance comparisons with attention to validation design, reporting completeness, and clinical readiness rather than to assign formal risk-of-bias scores. THA: total hip arthroplasty; TKA: total knee arthroplasty; DCA: decision curve analysis; NR: not reported; AUC: area under the curve; EMR: electronic medical record; CV: cross-validation; NSQIP: National Surgical Quality Improvement Program; RF: Random Forest; SMOTE: Synthetic Minority Over-sampling Technique; PROBAST: Prediction model Risk Of Bias ASsessment Tool

Reference	Study (Author, Year)	Outcome	Data source	Validation	Missing data handling	Class imbalance	Calibration/Utility	PROBAST domains flagged	Notes
[13]	Huang et al., 2023	Surgical site infection	Ortho patients (train + external validation cohort)	External validation (separate external set)	NR	NR	Calibration + DCA reported	Analysis	Classic regression nomogram w/ calibration + DCA
[40]	Huang et al., 2021	Blood transfusion after THA/TKA	Multi-hospital EMR cohort	Random subsampling + 10-fold CV	Excluded missing/incorrect (0.73%)	NR	NR	Analysis	Reports AUC-focused comparison; calibration not highlighted
[47]	Zang et al., 2024	Perioperative blood transfusion (hip surgery)	Retrospective hip surgery cohort	Temporal split (first 70% train / last 30% test)	Excluded missing data	NR	Calibration + Brier + DCA reported	Analysis	Time split is stronger than random split; still single-system retrospective
[59]	Jiang et al., 2024	Pedicle screw loosening	Lumbar fixation cohort	Random split (8:2) + 10-fold CV	Imputed (<20%) w/ RF regression; otherwise excluded	NR	Calibration plots + Brier	Analysis	Strong reporting on calibration/Brier; still internal validation only
[60]	Xiong et al., 2023	Spine outcome model; per paper	Spine surgery imaging/clinical cohort	Train/validation split (0.75/0.25)	Excluded incomplete imaging	NR	Calibration + DCA reported	Participants / Analysis	Imaging exclusion can introduce selection bias; no external validation shown
[61]	Chen et al., 2023	Surgical site infection prediction	Retrospective cohort	Train/test split (reported)	Excluded incomplete clinical data	NR	Calibration curves reported	Analysis	Calibration reported, but imbalance handling not clearly described
[62]	Zhang et al., 2024	SSI following spine surgery	986 pts “complete data” cohort	5-fold CV (4 folds train / 1 validate)	Complete-case (“complete data” only)	NR	NR	Participants / Analysis	Internal CV only; AUC-heavy evaluation; missing handling = exclusion
[71]	Gupta et al., 2025	30-day mortality (femoral shaft fracture surgery)	NSQIP dataset	Stratified 80/20 split + 10-fold CV	Dropped vars >5% missing; kNN imputation for remaining	SMOTE + Tomek	Calibration slope/intercept + Brier	Analysis	Best reported “methods hygiene” among these (imbalance + calibration + imputation)
[72]	Han et al., 2025	Postoperative infection (nosocomial infection paper)	Retrospective cohort (2011–2024)	Random 70/30 split + 10-fold CV	Imputation reported (details in supplement)	NR	Brier reported	Analysis	Strong model-development description; still internal validation only

Validation & Generalizability Challenges

A. Overfitting in single-institution studies: Single-institution studies that develop AI models to predict postoperative complications in orthopedics often fall prey to overfitting, where the model performs exceptionally well on its original dataset but fails to generalize to new patient groups. This occurs because the algorithms inadvertently learn local trends in patient demographics, surgical techniques, clinical workflows, and documentation practices, rather than universal predictive factors. Moreover, because the postoperative complications being investigated are relatively rare events, the underlying prevalence in most cohorts is low, which inherently limits the PPV of these models even when overall performance metrics appear strong. For example, a systematic review found that about 75% of perioperative AI models were built using data from a single center, with only 13% undergoing external validation, which raises serious concerns about their applicability elsewhere [9]. Similarly, a multi-institution framework showed that models trained on a single-center dataset had limitations when applied to other hospitals with different case mixes, workflow patterns, and documentation style [76].

To reduce the risk of overfitting, it is important to validate AI models across multiple institutions rather than relying on one hospital’s data. For example, one large-scale study from 2022 developed an AI model to predict patient mortality within 30 days after surgery, where researchers included data from four separate hospitals and applied rigorous statistical methods, such as cross-validation and bootstrapping, to ensure the model was reliable across diverse patient groups [77]. Their approach resulted in strong performance, achieving AUROC values of about 0.94 both within their original hospitals and when tested externally. A 2025 review emphasizes that combining feature selection, regularization techniques, and rigorous cross-validation significantly improves the generalizability of AI models in orthopedic applications, reducing the risk of overfitting and enhancing robustness to diverse clinical data [78]. By applying these safeguards and validating models across diverse patient populations, researchers can help ensure AI tools are both trustworthy and ready for real-world use in orthopedics.

B. Lack of multicenter validations: One of the most critical barriers to bringing AI into routine orthopedic care is the lack of multicenter validations. Most models are developed and tested at a single institution, which often leads to inflated performance metrics that fail when applied elsewhere due to differences in patient demographics, hospital workflows, or surgical techniques [79,80]. Aydın and Orhan, in their 2025 systematic review, noted that many orthopedic AI studies rely heavily on retrospective, unicentric datasets and rarely perform external validation, which casts doubt on their real-world reliability [80]. In contrast, Lee et al.'s multi‑center study involving four independent hospitals developed a 30‑day postoperative mortality model using just 12-18 preoperative variables and reported AUROC values of ~ 0.94 across both internal and external cohorts, demonstrating strong generalizability [77]. Similarly, an AI platform designed to predict postoperative ambulatory ability in spinal metastasis patients was validated in two external hospital cohorts, achieving AUCs of 0.88 and 0.92, which further supports multi‑institutional robustness [81]. Pooling data from multiple centers allows researchers to identify hidden biases and improve the fairness, reliability, and generalizability of AI models in clinical practice. Addressing this gap in validation is not only important for ensuring methodological rigor but also crucial for building the clinical confidence and trust necessary for the safe and effective adoption of AI tools in patient care.

C. Data bias: One of the key challenges in developing AI models for predicting postoperative complications in orthopedics is the presence of data bias, particularly when datasets are imbalanced or lack diversity. When minority populations, such as certain racial or socioeconomic groups, are underrepresented in training data, AI algorithms may produce less accurate predictions for these groups, perpetuating existing healthcare disparities. For example, studies have shown that AI models trained predominantly on data from White patients tend to have reduced accuracy when applied to racially diverse populations, potentially leading to unequal care recommendations or risk assessments [82]. When certain groups, such as racial minorities or people from lower socioeconomic backgrounds, are left out or underrepresented in the data used to train AI models, the predictions made for these groups can be less accurate. This means that patients who are already at risk of receiving unequal care could face even greater disadvantages, which goes against the goal of providing fair and equitable healthcare for everyone.

To address this issue, researchers must prioritize the inclusion of diverse populations in AI model development and validation. Practical strategies include balanced sampling, bias-aware algorithm design, and subgroup performance reporting to ensure models perform equitably across different demographic groups [83]. Another review noted that failure to address data imbalance could undermine both predictive performance and ethical implementation in orthopedic care [84]. This reinforces the need for AI systems that not only improve outcomes but do so fairly and responsibly across all patient populations.

Barriers to Clinical Implementation

A. Integration with EHRs: Successfully integrating AI tools into existing EHR systems is a complex but critical step for their adoption in orthopedic practice. Research has shown that AI models are often developed in isolation from hospital infrastructures, creating difficulties when attempting a real-time connection between algorithms and live patient data [85]. Without seamless interoperability, clinicians may experience delays or disruptions by only receiving AI-driven insights after completing clinical workflows, which limits their utility and may even pose safety concerns. Moreover, hospitals vary widely in data formats, coding frameworks (e.g., HL7, FHIR), and documentation standards, making it challenging to deploy a single solution across multiple sites [86].

In addition to technical integration, another important barrier is workflow adaptation. Studies emphasize that without thoughtful interface design and the integration of AI seamlessly into existing provider workflows, adoption remains low [87]. User-centered approaches, such as embedding AI alerts within routine clinical screens and providing training, are key to making these tools helpful rather than disruptive [88]. In summary, achieving interoperability, standardizing data pipelines, and aligning AI outputs with clinician needs through adaptive workflow design are crucial steps toward safely implementing AI in orthopedic care.

B. Explainability and clinician trust: Many advanced AI models used in orthopedics are “black-box” systems whose internal logic remains opaque, making it difficult for clinicians to understand or verify the reasoning behind predictions [85]. This lack of explainability makes it challenging for healthcare providers to trust AI outputs, as they cannot easily determine whether recommendations are based on clinically relevant factors or spurious correlations [85]. Studies have demonstrated that explainable AI (XAI) techniques, such as using saliency maps, Shapley values, and rule-based models, can significantly enhance the interpretability of AI outputs [89]. Importantly, systematic reviews have shown that XAI tools can increase clinician trust when the explanations provided are clear, concise, and contextually relevant [90]. Without clear explanations behind AI predictions, many healthcare providers may hesitate to use these tools in their decision-making, worried about unpredictable outcomes or potential legal risks. Making AI systems more transparent and easier to understand is key to earning clinicians’ trust and ensuring these technologies can be safely and responsibly integrated into everyday orthopedic care.

C. Regulatory and medico-legal considerations: The integration of AI-driven prediction tools into orthopedic practice faces significant challenges due to unclear regulatory pathways and medico-legal concerns [91]. Currently, there is a lack of standardized guidelines for the development, validation, and approval of these tools, creating uncertainty for both developers and healthcare providers [92]. Without clear regulatory oversight, it becomes difficult to ensure the safety, efficacy, and ethical use of AI in predicting postoperative complications [93]. Moreover, liability concerns arise when AI-generated recommendations influence clinical decisions, raising questions about accountability in cases of adverse outcomes [94]. These legal uncertainties make many clinicians hesitant to trust or rely on AI technologies, which in turn slows down their use in orthopedic surgery. To facilitate clinical integration, there is a pressing need for regulatory bodies to establish comprehensive frameworks that address validation, transparency, and shared responsibility between AI developers and medical professionals [95].

Future Directions and Research Gaps

To fully realize the potential of AI in predicting postoperative complications in orthopedics, future research must address current limitations while exploring innovative approaches. One promising strategy is federated learning, which allows AI models to be trained across multiple decentralized healthcare data sources without the need to directly share sensitive patient information [96]. This approach not only protects patient privacy but also helps create models that are more robust and generalizable across diverse clinical settings [97]. Additionally, research should focus on creating AI models that can evolve by incorporating new clinical data and adjusting to shifts in patient populations and treatment practices. Such dynamic models can help maintain accuracy and relevance, preventing the stagnation that can occur with existing static algorithms [98].

Another important direction lies in fostering closer collaboration between AI systems and healthcare professionals. Rather than relying solely on algorithmic outputs, the development of hybrid models, which combine the predictive power of AI with the nuanced judgment of experienced clinicians, could enhance both trust and effectiveness [99]. These models are designed to offer clear, tailored guidance that takes into account the unique details of each patient’s situation, helping clinicians make informed decisions without losing sight of the complexity of real-world care. Ultimately, balancing AI with human expertise may be the key to ensuring that AI tools serve as valuable assistants rather than replacements. While these findings summarize current AI performance across orthopedic outcomes, persistent gaps in real-world adoption, equity, and workflow integration motivate the proposed strategies and the VIR framework for responsible implementation we bring forward in the Discussion.

Discussion

AI is rapidly transforming the landscape of orthopedic surgery by enabling more precise, personalized risk prediction for postoperative complications. Current applications in total joint arthroplasty, spine surgery, and trauma care demonstrate that AI models can outperform traditional statistical methods in predicting infections, venous thromboembolism, transfusion needs, and LOS. However, despite these advancements, significant challenges remain before AI can be fully integrated into everyday orthopedic practice. Issues such as overfitting in single-center studies, lack of external validation, data bias, and the difficulty of integrating AI into electronic health records continue to limit real-world adoption. Additionally, the “black-box” nature of many AI models creates skepticism among clinicians, who require transparency and explainability to trust AI-assisted recommendations. Consistent with broader evaluations of AI in clinical medicine, the strengths, weaknesses, opportunities, and threats (SWOT)-based analyses emphasize that sustainable impact depends on ethically governed, transparent, and safety-focused implementation strategies [100]. As detailed earlier, apparent performance differences across model families (e.g., logistic regression vs Random Forest/boosted trees vs neural networks) are often driven by data modality, event prevalence, and validation rigor rather than an “AI” label, an algorithm-aware perspective that is used to interpret comparative findings. A critical implication of this review is that comparative conclusions should be interpreted through an algorithm- and methodology-aware lens: boosted trees may be preferred for many tabular perioperative prediction tasks, whereas neural networks may add value primarily when multimodal inputs are available. However, higher AUC alone is insufficient for adoption; models intended for clinical deployment should demonstrate calibration at clinically relevant thresholds and report utility-focused metrics (e.g., decision-curve net benefit), particularly for rare complications where discrimination can remain high while positive predictive value is modest.

To overcome these barriers and facilitate responsible AI integration, future research should prioritize strategies that enhance generalizability, transparency, and clinical relevance. Federated learning presents a promising solution by allowing models to be trained across multiple institutions without compromising patient privacy, thus improving model robustness. The development of continuously updating AI models that evolve with new clinical data can help maintain accuracy in the face of shifting surgical practices and patient demographics. Equally important is the creation of hybrid decision-making frameworks that combine AI predictions with clinician expertise, ensuring that AI serves as an aid rather than a replacement for human judgment. Building trust through explainable AI, rigorous multicenter validations, and clear regulatory pathways will be essential to unlocking the full potential of AI in orthopedic care, ultimately leading to safer surgeries, better patient outcomes, and more efficient healthcare delivery. These themes mirror the earlier sections on validation, generalizability, data bias, and implementation barriers, and here we synthesize them into a pragmatic roadmap rather than repeat the detailed methodological considerations.

As demonstrated in Table 1, our synthesis provides one of the first direct comparative frameworks between AI-based models and traditional regression approaches in orthopedic surgery. By presenting reported AUC ranges across outcomes drawn from primary studies, this review moves beyond a purely descriptive catalog and offers a structured comparative synthesis suggesting that AI/ML models often achieve higher discrimination in many orthopedic prediction tasks, while highlighting substantial limitations in validation rigor, calibration reporting, and bedside utility. Building on this synthesis, the novelty of this review lies not only in pooling comparative performance data across orthopedic domains but also in explicitly contrasting AI against regression and proposing the VIR framework to guide clinical translation. 

To ensure AI improves rather than disrupts orthopedic care, we highlight three pragmatic strategies: (i) hybrid clinician-AI workflows that pair individualized model predictions with surgical judgment, (ii) federated learning to overcome single-institution bias by training across distributed registries without sharing raw patient data, and (iii) health-equity safeguards, balanced sampling, bias audits, and subgroup performance reporting, to avoid amplifying disparities and to maintain fairness alongside accuracy.

Proposed VIR Framework: Validation, Integration, and Regulation

To consolidate these insights, we propose a structured roadmap for integrating AI into orthopedic surgery, summarized as the VIR framework: Validation, Integration, and Regulation. Validation requires rigorous multicenter testing to ensure predictive models are generalizable across diverse institutions, patient demographics, and surgical practices. Integration emphasizes seamless incorporation of AI into electronic health record systems and clinical workflows, supported by user-centered interface design to maximize adoption. Regulation highlights the urgent need for transparent standards, medico-legal clarity, and shared accountability between developers and clinicians to ensure safety and trust. Together, these three pillars provide a pragmatic and actionable guide for translating AI innovations from research into everyday orthopedic practice, addressing not only performance but also equity, usability, and accountability. 

Looking ahead, several near-term priorities emerge for translating these insights into practice. Prospective multicenter studies that leverage federated or other privacy-preserving designs are needed to rigorously stress-test generalizability across diverse populations. Workflow-embedded tools, such as dashboards or order-set prompts, should be co-designed with clinicians to ensure that AI predictions are delivered at the point of decision-making rather than as stand-alone outputs. Finally, routine equity monitoring must be incorporated into model evaluation, with predefined subgroup performance metrics and corrective retraining strategies applied when bias or model drift is detected. Together, these steps can strengthen trust, enhance clinical utility, and support safe integration of AI into everyday orthopedic practice.

Limitations

Finally, several limitations should be acknowledged. This narrative review synthesized heterogeneous study designs, endpoints, and validation strategies across arthroplasty, spine, and trauma, which complicates direct quantitative pooling. A further limitation of both the existing literature and our synthesis is that most orthopedic AI studies prioritize AUC while incompletely reporting other metrics such as sensitivity, specificity, likelihood ratios, F1 score, and decision-curve net benefit. As a result, it remains uncertain whether AI models provide clinically meaningful gains over well-constructed regression tools at clinically relevant decision thresholds. Reported AUC ranges may also reflect publication bias and institutional case-mix effects. We emphasized peer-reviewed studies with internal or external validation where available, but the field still lacks large, prospective, multicenter evaluations that would allow meta-analytic effect size estimates. 

## Conclusions

AI holds great promise for improving the prediction of postoperative complications in orthopedic surgery. By synthesizing comparative evidence, this review demonstrates that AI models often outperform traditional regression approaches, with reported AUC values typically in the 0.80-0.90 range for AI/ML models and 0.60-0.75 for classical methods across the studies reviewed. However, these comparisons should be interpreted in light of heterogeneity in datasets, endpoints, and validation design, and AUC alone may not capture bedside utility for low-prevalence events without calibration and threshold-based evaluation.

Beyond performance, we propose a pragmatic VIR framework: multicenter validation to ensure reliability, seamless EHR integration to support clinical workflows, and regulatory oversight to safeguard accountability. In addition, hybrid clinician-AI workflows and federated learning can maximize accuracy while protecting patient diversity and privacy. By explicitly addressing issues of bias, equity, and transparency, AI can evolve into a trustworthy adjunct that enhances perioperative planning, optimizes resource allocation, and improves patient outcomes in orthopedic surgery.
